# Diagnosis of *Clostridium difficile* infection by toxigenic culture and PCR assay

**Published:** 2018-10

**Authors:** Elnaze Zare Mirzaei, Mahdi Rajabnia, Farzin Sadeghi, Elaheh Ferdosi-Shahandashti, Mahmoud Sadeghi-Haddad-Zavareh, Soraya Khafri, Abolfazl Davoodabadi

**Affiliations:** 1 Infectious Diseases & Tropical Medicine Research Center, Babol University of Medical Sciences, Babol, Iran; 2 Department of Microbiology, Faculty of Medicine, Babol University of Medical Sciences, Babol, Iran; 3 Cellular and Molecular Biology Research Center, Health Research Institute, Babol University of Medical Sciences, Babol, Iran; 4 Infertility and Reproductive Health Research Center, Babol University of Medical Sciences, Babol, Iran

**Keywords:** Antibiotic associated diarrhea, *Clostridium difficile*, Polymerase chain reaction, Toxigenic culture

## Abstract

**Background and Objectives:**

*Clostridium difficile* is responsible for 15–25% of nosocomial antibiotic associated diarrhea (AAD) cases and all cases of pseudomembranous colitis. *C. difficile* has two major virulence factors, toxin A (enterotoxin) and toxin B (cytotoxin). The aim of this study was to determine the frequency of *C. difficile* strains in patients with diarrhea in Babol’ hospitals with toxigenic culture and PCR assay.

**Materials and Methods:**

One hundred stool specimens were taken from diarrheal patients in hospitals of the city of Babol. All patients had a history of antibiotic use. The samples were cultured on CCFA medium. In the next stage, toxigenic culture was performed for isolated *C. difficile* strains. Then, PCR assay was used to identify *gdh, tcdA* and *tcdB* genes among isolated *C. difficile* strains.

**Results:**

From the 100 stool samples, eight (8%) samples were positive in *C. difficile* culture. In toxigenic culture, two (2%) of these strains had cytopathic effects on Vero cells. All eight strains had the *gdh* gene. This gene is specific for *C. difficile*. Two strains that had cytopathic effects on toxigenic culture were positive for toxin genes.

**Conclusion:**

The frequency of toxigenic strains in different parts of the world is variable, and needs to be continually investigated. In the present study, the PCR method had a good correlation with toxigenic culture. Thus, it can replace the laborious and costly cell culture method.

## INTRODUCTION

*Clostridium difficile* is an important cause of nosocomial infections. Symptoms of *C. difficile* infection (CDI) range from asymptomatic carriage to mild diarrhea, colitis, severe life threatening pseudomembranous colitis and to fulminant colitis (1, 2). This microorganism is responsible for 15–25% of nosocomial antibiotic associated diarrhea (AAD) cases and all cases of pseudomembranous colitis (3, 4). The most predisposing factors for CDI include prior antibiotic therapy, age older than 65 years, and recent long-term hospitalization (5). *C. difficile* expresses two major virulence factors, which are toxin A (enterotoxin) and toxin B (cytotoxin) encoded via *tcdA* and *tcdB* genes respectively (6, 7).

There are various tests for diagnosis of CDI in laboratories. Some of these tests are enzyme immunoassay (EIA), glutamate dehydrogenase (GDH), cytotoxicity assay (CA), toxigenic culture and PCR. Enzyme immunoassay is a rapid method and is done directly on stool samples. Although this test is very fast, it has very low sensitivity (8). The GDH test detects glutamate dehydrogenase enzyme in the cell wall of *C. difficile*. GDH is considered a screening method because it is expressed by both the toxigenic and nontoxigenic strains. GDH is a very rapid, inexpensive and easy method. Unlike toxin A and B tests, this test has high sensitivity but low specificity. However, to confirm positive GDH test results, complementary tests are needed (9).

The cell culture cytotoxicity assay (CA), based on toxin B detection is the gold standard for diagnosis of CDI. However, this test is not routinely used by clinical microbiology laboratories, because it requires cell culture facilities and a reliable antitoxin for neutralization (10, 11). The PCR is used to detect toxin A or toxin B genes in strains or directly in fecal samples and in terms of sensitivity is similar to the cytotoxicity assay. In comparison to the cytotoxicity assay, PCR is a very fast method for the diagnosis of CDI (12). To the best of our knowledge, no study to date has examined *C. difficile* infection in patients with diarrhea in the hospitals of Babol. The aim of the present study was to determine the frequency of *C. difficile* infection in patients with diarrhea in the hospitals of Babol with toxigenic culture and PCR.

## MATERIALS AND METHODS

One hundred stool specimens were taken from diarrheal patients in hospitals of Babol. All patients had a history of antibiotic use, and demographics data of patients were collected via a questionnaire. The specimens were immediately transferred to the microbiology laboratory at Babol University of Medical Sciences. About one gram of stool specimen was suspended in a tube containing one mL of BHI broth (Merck, Germany) and one mL of ethanol 96% (ethanol shock) for 45 min. Then this suspension was cultured on CCFA (cycloserine-cefoxitin fructose agar; Merck, Germany) under anaerobic condition at 37°C for 72 h. The colonies which contained Gram-positive bacilli with 1–3 mm diameter, white to gray color, and horse odour were regarded as *C. difficile*. The isolated strains were stocked in BHI broth containing 15% glycerol and stored at −20°C.

### Toxigenic culture.

In the next stage, toxigenic culture was performed for isolated *C. difficile* strains. Vero cells were grown in a flask containing Dulbecco’s modified Eagle’s medium (DMEM; Gibco), 100 U/ml penicillin-streptomycin and 10% fetal bovine serum (FBS Gibco), and incubated at 37°C and 5% CO_2_ for 3–5 days. The cells were trypsinized and counted. About 10,000 cells were added to the wells of microtiterplate and were incubated at 37°C and 5% CO_2_ for 24 h to reach about 80% confluency.

*C. difficile* strains were cultured in BHI broth for 5–7 days at 37°C, then the culture medium was centrifuged (10 min at 1500 g). The obtaining supernatants were filtered (0.22 μm pore size), and 200 μl of filtrate was added to Vero cell culture (96-well microtiter plate; Biofil, China). The microtiter plate was incubated for 24–48 h at 37°C and 5% CO_2_. *C. difficile* strains which produce toxin (positive result), cause cytopathic effects in more than 50% of the cell monolayer. Supernatant obtained from a toxigenic *C. difficile* strain, which was previously isolated from a diarrheal patient, was used as a positive control in toxigenic culture test (13).

### Identification of *gdh, tcdA* and *tcdB* genes by PCR assay.

DNA extraction was performed by boiling methods (14). A single colony from every isolate was suspended in 50 ml of TES buffer (containing 50 mM Tris hydrochloride [pH 8.0], 5 mM EDTA, 50 mM NaCl), and the suspension was heated in a boiling water bath at 95°C for 10 min and centrifuged at 15,000 × g for 3 min. The resultant supernatant was used as DNA template. Extracted DNA was stored at −20°C. *C. difficile* and its toxins were identified by PCR method targeting the *gdh* (glutamate dehydrogenase), *tcdA* and *tcdB* genes. The primers used in this study are listed in Table 1.

**Table 1.** Primers used in this study.

**Genes**	**Primer name**	**Sequence (5′–3′)**	**Amplicon size (bp)**	**References**
*gdh*	*gdh R*	CTGATTTACACCATTCAGCCATAGC	736	(15)
	*gdh F*	GGAAAAGATGTAAATGTCTTCGAGATG		
*tcdA*	*tcdA*-F3345	GCATGATAAGGCAACTTCAGTGGTA	629	(16)
	*tcdA*-R3969	AGTTCCTCCTGCTCCATCAAATG		
*tcdB*	*tcdB*-R6079A	GCATTTCTCCATTCTCAGCAAAGTA	410	(16)
	*tcdB*-F5670	CCAAARTGGAGTGTTACAAACAGGTG		

For each gene, the PCR was run in 20 μL reaction mixture containing 10 μL master mix PCR, 2 μL DNA template, 20 pmol of each primer and 6.4 μL PCR grade water. PCR was performed in a thermocycler (A & E, England) using the following conditions: 5 min at 95°C, followed by 30 cycles of 1 min s at 94°C, 1 min at 54°C for *gdh* gene, 1 min at 56°C for toxin genes, 1 min at 72°C, and a final extension of 10 min at 72°C. The presence of each gene was determined by electrophoresis on a 1.5% agarose gel. In each PCR run, DNA template from a toxigenic *C. difficile* and water were used as positive and negative controls, respectively.

## RESULTS

**Fig. 1.**
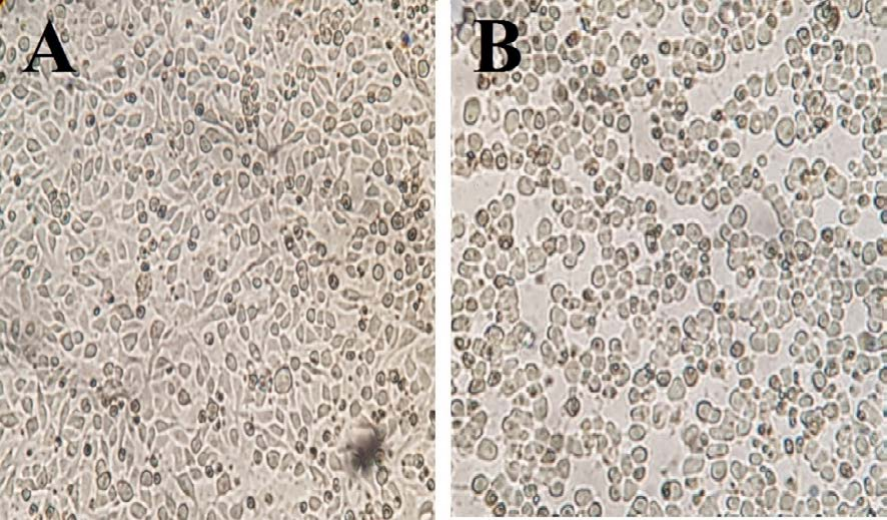
Cytopathic effects (CPE) of *C. difficile* supernatant on Vero cells. A; Toxin negative *C. difficile* B; Toxin positive *C. difficile*. CPE: Toxins deform Vero cells from spindle form to round form in more than 50% of the cells.

From the 100 patients with diarrhea, 45 (45%) cases were males and 55 (55%) cases were females. The stool samples were obtained from ICU ward (62%), infectious ward (15%) and other wards including respiratory, hematology, neurology, gastroenterology and internal medicine (23%). From the 100 stool samples, eight (8%) samples were positive in *C. difficile* culture. In toxigenic culture, two (2%) of these strains, had cytopathic effects (CPE) on Vero cells (Fig. 1). Cytopathic effects of *C. difficile* toxins on spindle form Vero cells characterized by rounding up these cells. Demographic data of eight patients with positive *C. difficile* culture is shown in Table 2. A toxigenic *C. difficile* strain was isolated from a woman in infectious ward, and another toxigenic strain isolated from a man in ICU ward.

**Table 2.** Demographic data of eight patients with positive *C. difficile* culture.

**Male / Female**	**Age**	**Hospital ward**	**Length of admission (days)**	**Antibiotic used**	**Toxigenic culture**	**Gene**
F	23	Infectious	10	Clindamycin Ceftriaxone	+	*gdh, tcdB*
M	49	ICU	16	Levofloxacin	+	*gdh, tcdA, tcdB*
F	72	ICU	8	Ceftriaxone	-	*gdh*
F	80	ICU	44	Ciprofloxacin- Cefepime	-	*gdh*
F	77	ICU	20	Meropenem- Ciprofloxacin	-	*gdh*
F	79	ICU	10	Meropenem-Vancomycin- Ciprofloxacin	-	*gdh*
F	35	ICU	12	Meropenem-Levofloxacin-Nitromicin Amphotericin-Fluconazole-Cotrimoxazole	-	*gdh*
M	80	ICU	47	Cefepime-Colistin-Levofloxacin Erythromycin-Fluconazole	-	*gdh*

**Fig. 2.**
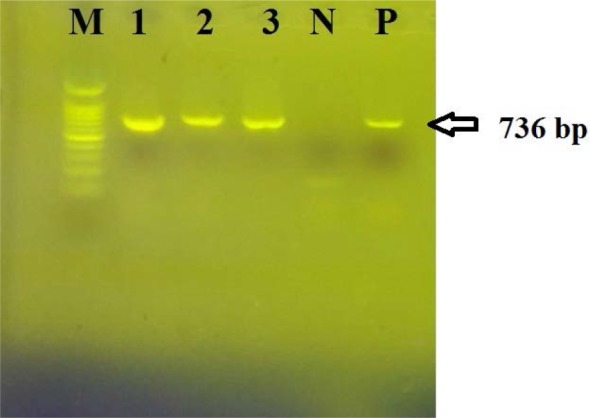
PCR products of the *gdh* gene. M: DNA size marker, 100 base pair, Lanes1, 2, and 3: PCR products of the *gdh* gene (736 bp) for three *C. difficile* strains. P: Positive control sample, N: Negative control sample.

**Fig. 3.**
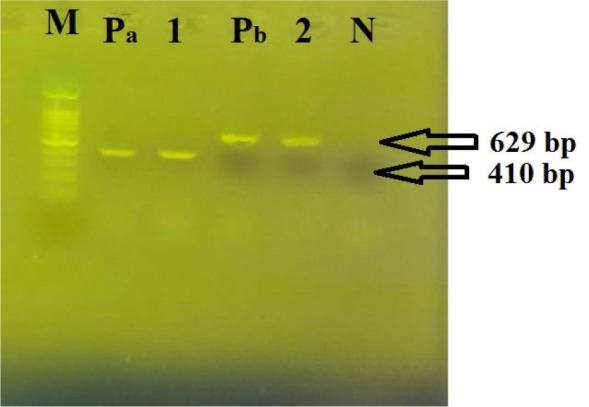
PCR products of *tcdB* and *tcdA* genes. M: DNA size marker, 100 base pair, Lane1: PCR product of *tcdB* gene (410 bp), 2: PCR product of *tcdA* gene (629 bp), Pa: positive control for *tcdB* gene, Pb: positive control for *tcdA* gene. N: Negative control sample.

All eight strains had the *gdh* gene (Fig. 2). This gene is specific for *C. difficile* (2). Among these eight strains, two strains that previously had cytopathic effects on toxigenic culture were positive for toxin genes. The strain isolated from the feces of the 49-year-old man was positive for *tcdA* and *tcdB* genes, and the strain isolated from the feces of a 23-year-old female patient was positive for the *tcdB* gene only (Fig. 3, Table 2).

## DISCUSSION

Infections with *C. difficile* have significantly increased over the past two decades (17). Infections with this organism have been reported from Australia (5), European countries (18) and the United States (19). *C. difficile* infections usually occur after treatment with antibiotics in hospitalized patients. Antibiotics such as β-lactams and clindamycin decrease the normal flora of the intestine and ultimately create the condition for further growth of *C. difficile* in the intestine as well as the development of clinical symptoms associated with this infection (20, 21). The pathogenicity of this bacterium is related to two major virulence factors of enterotoxin A and cytotoxin B (22, 23). Strains that have the ability to only produce toxin B are clinically important (24, 25).

This study for the first time examined the frequency of toxigenic *C. difficile* strains in hospitalized patients in Babol hospitals. The frequency of toxin positive *C. difficile* among diarrheal patients by toxigenic culture and PCR was 2%. In 2016, Lotfian et al. studied 171 samples of suspected cases of diarrhea associated with *C. difficile* in Tehran. Their results showed that 10 (5.8%) samples were positive with both PCR and toxigenic culture. Of these 10 strains, 8 strains were *tcd*A + B + and 2 strains were *tcd*A–B +. They found very good agreement between toxigenic culture and PCR. Our study, similar to Lotfian et al., showed that PCR has good agreement with the toxigenic culture method (26).

Another study by Sadeghifard et al. in 2004 was carried out on stool specimens from patients with diarrhea in Tehran hospitals. In their report, the prevalence of toxin producing *C. difficile* by the cytotoxicity method was 6.1% (27). Azizi et al., in 2011, studied 98 diarrheal patients in Tehran and reported 39.8% (39) samples as *C. difficile* culture-positive. Among 39 *C. difficile* strains, 15 (15.3%) strains were positive for toxin genes, 12 (12.2%) strains had toxin B and A, two (2%) strains had only toxin A (A+B−) and one (1%) strains had only toxin B (A–B+) (28). In another study in Iran, conducted by Goodarzi et al. in 2012, 108 patients with diarrhea were studied at Taleghani Hospital in Tehran. From 108 patients, 17 (15.7%) toxigenic strains were isolated. Among the 17 strains, four (23.9%) strains had only toxin B (AB+), one (5.9%) strain had only toxin A (A+B−), and 12 (70%) strains had both toxins (A+B+) (29).

In the present study, frequency of toxigenic strains of *C. difficile* isolated from the patients with diarrhea in Babol was low (2%). In the study by Sadeghifard et al. and Lotfian et al., similar to the present study, the prevalence of toxigenic strains of *C. difficile* was reported low (6.1% and 5.8%, respectively) (30).

Prevalence of diarrhea in a Turkish hospital was reported 7% of all hospital infections and *C. difficile* was isolated from 18.2% of hospitalized patients with diarrhea (31). In a study by Garcia et al., which was conducted in Brazil, prevalence of toxigenic strains of *C. difficile* in diarrheal patients was reported at 13.8% (32). Sachu et al., in 2018, studied 660 patients with AAD in India, and they identified *C. difficile* infection in 9.7% patients by NAAT (33). The prevalence of toxigenic strains of *C. difficile* in other countries like India (34), Indonesia (35), and Germany (36) were reported as 4%, 5.6%, and 11.1%, respectively.

Due to geographical changes, diarrhea accounts for 1–14% of all hospital infections worldwide (30). Prevalence of *C. difficile* diarrhea differ according to the population of different hospitals and is affected by predisposing factors such as age, type and duration of antibiotic use, severity of underlying diseases and duration of admission (37, 38). In this study, for the first time *C. difficile* infection was identified in patients with diarrhea in Babol hospitals. PCR assay had good correlation with toxigenic culture, therefore, it can replace the laborious and costly cell culture method.
